# Supplementary material for: The interactome of intact mitochondria by cross-linking mass spectrometry provides evidence for coexisting respiratory supercomplexes
Source: Mol Cell Proteomics. 2017 Dec 8;17(2):216–32. doi: 10.1074/mcp.RA117.000470 (PMC5795388; doi:10.1074/mcp.RA117.000470)
Supplement: Supplemental Data [file supp_RA117.000470_133922_0_supp_23978_fzffwf.zip › spectra_annotation/mito_DR_spectra_annotation/396-1-3-1-22-1.pdf]

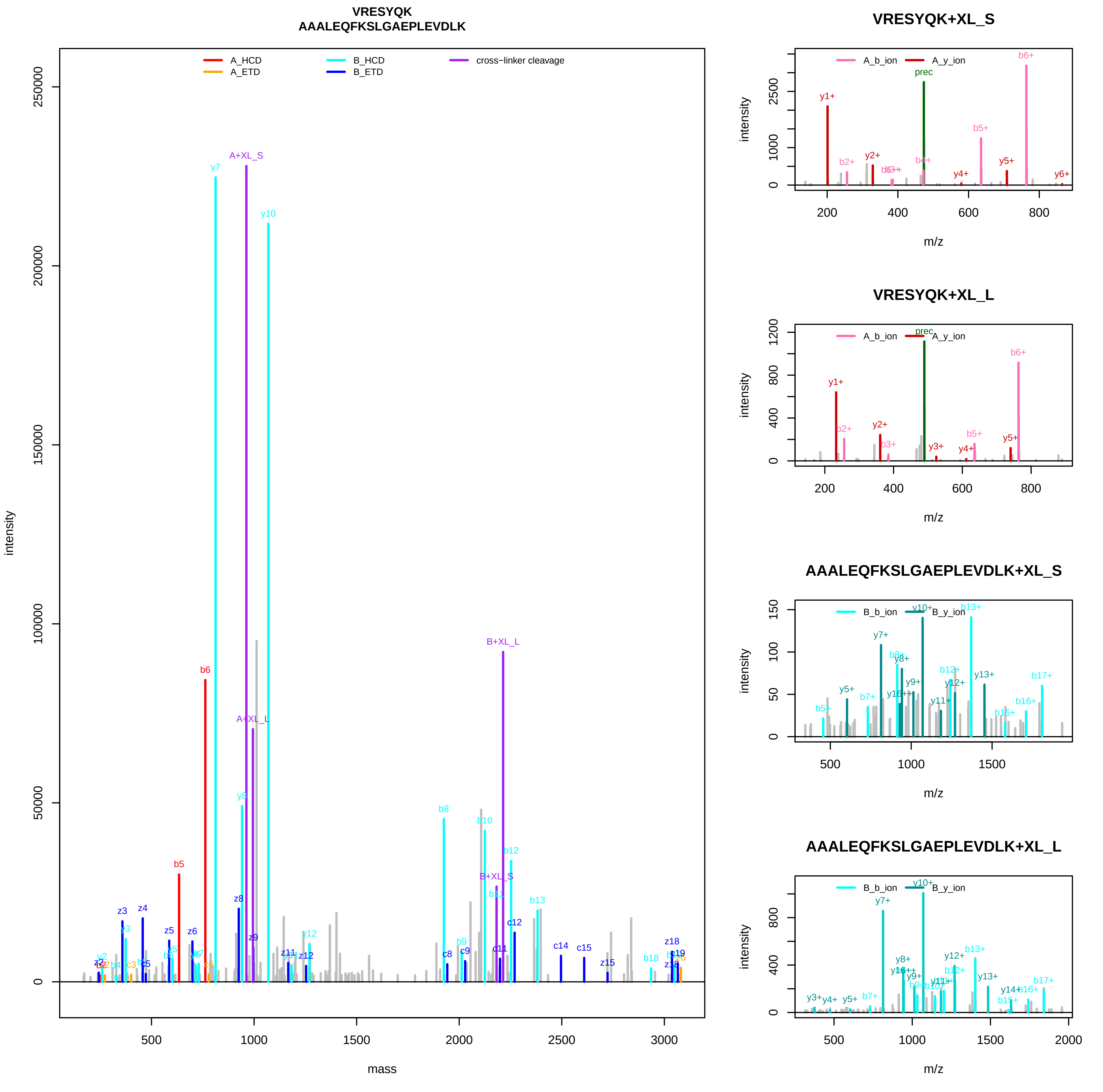
VRESYQK
AAALEQFKSLGAEPLEVDLK
A_HCD
A_ETD
B_HCD
B_ETD
cross−linker cleavage
intensity
mass
0
50000
100000
150000
200000
250000
500
1000
1500
2000
2500
3000
A+XL_S
A+XL_L
B+XL_L
B+XL_S
y7
y10
y8
b6
b5
b8
b10
b12
b13
z3
z4
z5
z6
z8
z9
c8
c9
c11
c12
c14
c15
z15
z18
c19
b18
y12
z11
z12
VRESYQK+XL_S
VRESYQK+XL_L
AAALEQFKSLGAEPLEVDLK+XL_S
AAALEQFKSLGAEPLEVDLK+XL_L
A_b_ion
A_y_ion
B_b_ion
B_y_ion
prec
m/z
intensity
